# Report on Indigenous Botany

**Published:** 1867-05

**Authors:** 

**Affiliations:** Grandview, Ill.


### ARTICLE XXI.

### REPORT ON INDIGENOUS BOTANY.

BY —— MASSEY, M. D.

Read before the Esculapian Society, October, 1866.

MR. PRESIDENT AND FELLOWS OF THE ESCULAPIAN SOCIETY:

*Gentlemen,*—As chairman of your committee on Indigenous Botany, I feel it my duty to present a report—a task however which I feel myself utterly incompetent to perform under the most favorable circumstances. And when I remind you of the very elaborate and interesting report of my highly esteemed friend Dr. Miller on this subject, at the last meeting of this society, I cannot fail to excite your deepest sympathies and commiseration on my behalf.

It will not therefore, be my aim to take a very extensive survey of this field of enquiry, but I shall confine my remarks to but a few species of a single genus of the flora of our beautiful country.

The first to which I shall call your attention is the Asclepias Cornuti.

Asclepias is from the Greek of Esculapius, fabulous god of medicine and physicians. It belongs to the natural order of Asclepiadaceæ, and all of its species as far as I know, to Pentandria Digynia of the artificial system of Linnæus.

The distinguishing characteristics of this genus are that the leaflets are cucullate or hooded, with an averted horn-like process at the base, curved toward the stigma.

Six species I know from observation, to be indigenous to this State and County, namely, A. Cornuti, A. Tuberosa, A. Incarnata, A. Purpurasceus, A. Obtusifolia, and A. Sullivantii. The first three of which are ranked in the secondary list of medicinal substances in the U. S. Dispensatory.

The species under consideration is the A. Syriaca of the U. S. Dispensary, or common silk-weed, a very common, coarse, lactescent perennial plant, growing along lanes and hedges, preferring a rich sandy soil.

The stem is simple, stout, from three to five feet high; leaves oblong-ovate, short-petiolate, mucronate, downy beneath, pedicels shorter than the leaves, densely many-flowered; hoods of crown ovate obtuse, not longer than the uncinate horn; corolla lobes ovate, reflexed; leaves five to eight inches long, by two to three inches wide; veinlets at nearly right angles with mid-vein; peduncles stout, between petioles, bearing a globular umbel of about a hundred greenish purple flowers, few of which prove fruitful; folicles sprinkled with soft warty spines, full of seeds with their long pappus or silk.

It flowers in July and August. The stem and leaves give out a milky juice when wounded; hence one of its names, milk-weed.

This juice has a sub-acrid taste and an acid reaction. Dr. C. List found the chief solid ingredient of the juice to be a peculiar crystaline substance of a resinous character, closely allied to lactucone, and which he proposes to call asclepione.

The juice is coagulated by heat, filtered, then digested with ether, which dissolves the asclepione and yields it by evaporation.

Dr. Shultz found 80 parts of the juice to contain 69 of water, 3.5 of a wax-like fatty matter, 5 of caoutchouc, and 0.5 of gum, 1 of sugar with salts and acetic acid, and 1 of other salts.

It is highly probable that the asclepione of Dr. List is the aggregate of the fatty and gummy matter of the juice soluble in ether, as nine parts out of eleven were shown to be of this character by Schultz.

These analyses throw little or no light on the medicinal properties of the juice, if indeed it is possessed of any.

The root however is the medicinal part, the juice of which is not milky. Dr. Richardson of Mass. found the root to possess anodyne properties. He gave it with advantage to an asthmatic patient, and in a case of typhus fever attended with catarrh. In both instances it appeared to promote expectoration, to relieve pain, cough and dyspnœa.

He gave a drachm of the powdered bark of the root in divided doses during the day, and employed it also in strong infusion.

Dr. Thomas, of Rocky Springs, Miss., stated in a letter to one of the authors of the U. S. Dispensatory, that he had employed the root in Scrofula with great success, and in Dyspepsia with advantage. He found it cathartic and alterative, but observed no anodyne property. He was induced to try it, having noticed that it was much used by the planters in scrofula and other diseases; and by the recommendation of Dr. McLean of Kentucky, who had employed it for twenty years in scrofula with most satisfactory results.

Dr. McLean also speaks very highly of it as an alterative in hepatic affections. He is however of opinion that it might be possible that he was using a different species from that described in the U. S. Dispensatory.

The doubt expressed by Dr. McLean, as to the species used by him might, at first, seem to invalidate his testimony in regard to the virtues of this species of asclepias; but when we remark the different appearance of different specimens, grown in different soils—and at the same time the impossibility of confounding it with any other known species—we are inclined to the opinion that Dr. McLean was confused by the first of the above facts, and that he was using the asclepias cornuti.

Dr. R. S. CANTHORN, of Richmond, Va., used it successfully in several cases of intermittent fever; and it is considered by him a valuable anti-periodic; he gave from four to six grains of the powdered bark, in form of pill, every two or three hours, augmenting the quantity to three times that dose, two or three times before the paroxysm.

I cannot speak as to its virtues as an anti-periodic, never having used it to fulfill such an indication; but as an alterative in scrofula, and as a gentle tonic in dyspepsia, I believe it to be a valuable remedy, and worthy of the attention of the profession.

I think it may reasonably be expected to do good in cachectic diseases, generally attended with torpor of the bowels, which it has a tendency to regulate; this, I believe it effects, in part at least, by giving tone to and stimulating the muscular coat, and in part by exciting a flow of bile, and other secretions, into the alimentary canal.

I have used it in strong decoctions; half ounce of the dried root to one pint of water—to be taken during the course of the day, and continued for a considerable period of time, unless it acts too much upon the bowels, in which case the dose should be reduced.

I next call your attention to asclepias tuberosa; this is the butterfly weed, or pleurisy root; it differs from all other species of asclepias, in not possessing a milky juice.

The stem is ascending, hairy, with spreading branches at the top; leaves alternate, oblong-lanceolate, often sessile, umbels numerous, forming a large terminal corymb; hoods bright orange, oblong, narrow, with slender sub-falcate, sub-erect horns.

It inhabits dry fields and meadows in the Middle, Western, and Southern States.

The root is large, fleshy, tuberous, sending up numerous stems two feet high and very leafy; leaf at top only, quite sessile, acute; obtuse at base, two to four inches long, by half to one inch wide; petals and crown of equal length; follicles branco-late-pointed, containing long, silky down; it flowers in August.

There is a variety, growing mostly in the Southern States, the stem of which is decumbent, which has been raised to the rank of a species, by some botanists, under the name of A. Decumbens; the root is the medicinal part, which was brought into popular use by the Indians, who used it in a variety of diseases.

SHOEFF first brought it to the notice of the profession.

It is ranked, by Prof. DUNGLISON, as a stimulating diaphoretic. He, however, has but little to say about it, and appears to know less; as it certainly neither increases the heat of the surface nor accelerates the pulse.

I think that no one, who has any experience with it, will deny its powerful diaphoretic properties. It appears to act directly on the sudorific porous glandula of the skin; having no direct sedative action on the heart or vascular system; neither does it act by inducing nausea; for, although it is slightly nauseant in the recent state, it acts as certainly and as powerfully when dried.

It acts also as a cathartic, when given largely, especially in the recent state.

Dr. GRIFFITH says that it is a valuable remedy in a variety of diseases, especially of the respiratory organs, and that there is ample testimony of its beneficial effects, if properly administered, in this class of cases.

Dr. CHAPMAN states that it is very certain and permanent in its action, and is well suited to excite diaphoresis in the forming stages of inflammatory diseases.

It has been used with success in acute rheumatism; nor is it empirically prescribed in this painful disease; as our present knowledge of its pathology points plainly to the use of blood depurants and elimenatives; and when we take into account the fact, that the sudden suppression of the cutaneous transpiration is a most fruitful cause of this disease, we can easily comprehend how active diaphoretics are likely to prove peculiarly useful.

Dr. LOCKWOOD states that when used in strong decoction or infusion, in the proportion of one ounce to a quart of water, (a tea-cup full of which should be taken warm every three or four hours,) it acts with as much certainty as a diaphoretic, as jalap does as a cathartic.

It is also recommended, by Dr. EBERLE PARKER and others, in acute dysentery; and in cases where the skin is hot and dry, I believe it to be highly useful, in virtue of its diaphoretic properties; but I am inclined to the belief that it acts beneficially in this disease, independently of its sudorific properties—by, in some unknown way, modifying the action of the mucous membrane of the alimentary canal, as I believe it also does of the mucous lining of the bronché.

It is popularly esteemed in flatulent colic; hence one of its names, wind root; and I think there can be no doubt that it is gently tonic to the muscular coat of the stomach and bowels.

I prescribed it in one case of typhod fever in which the skin was hot and dry and considerable tympanitic distention of the bowels; the patient could not take oil of turpentine in any way, on account of idiosyncracy: a pint of the decoction, as prepared above, was given *ad libitum* through the course of the day, and I was highly pleased with its effects; its use was followed by relaxation of the skin, a subsidence of the tympanites, and a general amelioration of all the symptoms. Of course other adjuvants were used, but I attributed the subsidence of the tympanites, and the diaphoresis to the asclepias.

I would not be understood, however, to recommend it as a substitute for the oil of turpentine; especially where the alterative effects of the latter medicine on the ulcerated patches of the small intestines is desired; but cases may occur in which the oil cannot be administered, as that just narrated; under such circumstances, I would recommend the use of the remedy under consideration. Nor do I believe it to be entirely without altertive effects on the mucous membranes, as I have already hinted.

Dr. Lockwood speaks very highly of its use in promoting the eruption in the exanthematous fevers; that it is useful in promoting the eruption in measles, I can testify from observation; it is also useful in this disease on account of the pectoral symptoms that not unfrequently attend it.

Indeed, it is in diseases of the respiratory organs that it has enjoyed the highest reputation, as one of its names would indicate—pleurisy root.

Dr. L. K. Williams, of Ohio, my former preceptor, was much in the habit of prescribing it in pneumonia, especially the typhoid variety.

It appears to me to be much better adapted to the treatment of typhod pneumonia than ipicacuanha, on account of the depressing effects of the latter remedy; it is especially indicated when the skin is hot and dry.

Sulphate of quinia, opium, or one of the salts of morphia, with the decoction of asclepias tuberosa were the remedies relied on in this form of pneumonia by Dr. Williams, as they are by myself: under this treatment copious diaphoresis is speedily brought about, and should be encouraged until the disease begins to decline; of course I do not speak of other adjuvants and appliances, as I am not writing on pneumonia; but I have been thus particular in describing its use in this disease, as it is the one in which I would especially recommend it.

Dr. Lockwood advises to gather the root about the first of October, cut in transverse slices, dry in the shade, pulverize and bottle; as found in the shops it is reduced to a coarse powder, pressed into cakes and enveloped in paper, each book containing one pound.

Asclepias incarnate is the third and last species of asclepias that enjoys any reputation as a remedial agent.

The stem is tall, and branching above; leaves opposite, lanceolate, on short petioles, slightly tomentous; umbels numerous, erect, mostly terminal, often in opposite pairs; hoods ovate-oblong, with subfalcate ascending horns; found in wet places:

three to four feet high with two hairy lines; leaves from four to seven inches long, by one-half to one and a-half inches wide, rather abrupt at base, tapering to a very acute point on petioles one-half inch long; corolla, purple or flesh-colored; flowers in July and August.

The root is the medicinal part, and is esteemed by some to be analogous in remedial properties to asclepias syriaca. Dr. Griffith considers it a valuable cathartic and emetic; it has not, however, been sufficiently tested to determine its precise medicinal virtues.

Dr. TULLY, of New Haven, who has had an extensive experience with our indiginous remedies, says that it may be administered with advantage in catarrh, asthma, rheumatism, syphilis, and verminous affections.

The dose is from ½ drachm to a drachm of the powdered root: a more eligible form for administration is that of a fluid extract, which may be found in the shops; and is largely consumed by the Eclectic doctors who use it, as I am informed, in a variety of cachectic and glandular diseases in which an alterative impression is desired.

I have had no experience with it myself; and I should not have called your attention to it, but for the fact that it belongs to the genus under consideration, and has had such valuable properties attributed to it by those who have used it.

It grows in abundance in the swampy portions of this State; and any one who may desire to test its virtues may certainly do so by the aid of the botanical analysis above given, and the specimens I present you.

The other species above-named may or may not possess valuable remedial properties; a fact which can only be known by actual and repeated experiment; and, judging from the indisposition of the medical profession to verify the experiments of others, however encouraging they may be, it is not likely that we shall know until, by accident or otherwise, they are forced upon our attention.

My friend, Dr. Miller, in his report called the attention of this society to an anonymous plant, the virtues of which I have determined to test, upon a suitable opportunity presenting itself.

Believing that others were as favorably impressed as myself, will, I hope, be a sufficient apology for my giving the following botanical analysis:

I refer to verbena bractiosa.

Verbena or vervain (from the Celtic perfœn, to expel stone) belongs to the natural order verbenaceae: the species under consideration belongs to peutandria monogynia, of the artificial system: it is perennial; inhabits dry fields and roadsides of the Middle, Western, or Southern States.

It is decumbent, branched, very hairy, leaves lacinate rugous; spikes terminal, thick, many-flowered; bracts, lance-linear, longer than the flowers, thrice longer than the calyx; whole plant eight to ten inches long; leaves from one to two inches long; flowers small, blue, blooms from May to September.

Several species of verbena have enjoyed considerable popularity, both in this country and Europe.

Its popular name, vervain, shows that it was formerly held in high estimation for the expulsion of stone.

The verbena verticæfolia is esteemed by many as a powerful emmenagogue; the root being the part used in strong decotion.

My friend, Dr. Miller, used the verbena bractiosa with marked success in a case of scrofula with amenorrhœa. I understand him, however, to recommend it especially in scrofulous complaints.

Verbena hastata has also been used in scrofula: it is powerfully bitter, and may possess some tonic or alterative virtues which render it valuable in scrofulous diseases.

*Grandview, Ill., Oct. 31st, 1866.*

